# One-year outcome of manualised behavior therapy of chronic tic disorders in children and adolescents

**DOI:** 10.1186/s13034-021-00362-w

**Published:** 2021-02-20

**Authors:** J. B. Nissen, A. H. Carlsen, P. H. Thomsen

**Affiliations:** 1 Department of Child and Adolescent Psychiatry, Aarhus University Hospital, Aarhus, Denmark; 2 Institute of Clinical Medicine, Health, Aarhus University, Aarhus, Denmark

**Keywords:** Chronic tics disorder, Tourette syndrome, Children, Adolescent, Therapy, Long-term outcome

## Abstract

**Background:**

Chronic tic disorders are neurodevelopmental disorders that can be treated with Habit Reversal Training (HRT) and Exposure Response Prevention (ERP). Intermediate and long-term effects have been examined after individual treatment with HRT, whereas evaluation of long-term outcome after an initial treatment with ERP, or a combination of HRT and ERP is lacking.

The present study examines the long-term effect after a combined treatment with HRT and ERP delivered in an individual or a group setting

**Methods:**

Fifty-nine children and adolescents diagnosed with a chronic tic disorder were randomised to manualised treatment combining HRT and ERP as individual or group training. Forty-seven were re-examined 1 year after acute outcome. Outcome measures included Total Tic Severity score (TTS) measured by the Yale Global Tic Severity Scale (YGTSS) and Beliefs About Tics Scale (BATS)

**Results:**

In a mixed model, it was shown that the initial improvement with both individual and group treatment was maintained throughout the follow-up period. There were no significant differences between the two methods of treatment delivery. Of all participants completing the 12 months evaluation, 74.4% were considered responders. There was a significant positive association between the reduction of TTS and the reduction in BATS. In a latent class post-treatment trajectory analysis, two classes were identified, where high baseline severity increased the likelihood of being in the lesser responder class. Similar, but only as a trend, having ADHD, planning difficulties or hypersensitivity increased the risk of a lesser response.

**Conclusions:**

The present study compares the efficacy in individualised and group treatment of providing manualised therapy for child and adolescent tic disorders using two behavioural methods (combined HRT and ERP) both of which have been shown to have acute benefits but only one of which has been validated for longer term effectiveness. In the present study, both individualised and group treatments showed benefit throughout a 1-year follow-up period with several potential confounds affecting outcomes, while the relative benefits of either HRT and ERP were not addressed.

*Trial registration* NCT04594044, 1-10-72-216-15, registered 19th October 2020, retrospectively registered, https://register.clinicaltrials.gov/prs/app/template/Home.vm?uid=U0005BW2&ts=9&sid=S000ABEY&cx=-wlx7vb

The study is approved by the National Ethical Committee (1-10-72-216-15) and the Danish Data Protection Agency (1–16-02-490-15), registered 12 October 2015.

## Background

Chronic tic disorders are neurodevelopmental disorders characterized by motor and/or vocal tics. The course of the tic disorder may be very fluctuating both in intensity, symptom presentation and localisation [1–4].

Various therapeutic interventions are available for chronic tic disorder in children and adolescents which include Habit Reversal Training (HRT) and Exposure Response Prevention (ERP) [5].

Given the chronicity and waxing-waning time course of tic disorders, length of follow-up is an important consideration.

Several studies have examined the immediate or acute outcome of therapeutic treatment showing significant reductions in tic intensity [6–11]. Pringsheim et al. [12] published a comprehensive systematic review describing a “high confidence” that treatment with comprehensive behavioural intervention for tics (CBIT) was associated with a more pronounced reduction in tics intensity as compared to the effect of supportive therapy. They suggested further studies on long-term efficacy. The same group published practice guidelines for treating people with tics [13]. Comparably, Yu et al. [14] performed a meta-analysis showing a small to medium effect size for the efficacy of HRT. Some studies have further examined the intermediate (3–6 months) or long-term (10–12 months) follow-up of behaviour therapy in children, adolescents and adults.

The intermediate outcome of behaviour therapy for children, adolescents, and adults with chronic tic disorders was examined by Verdellen et al. [10] who re-evaluated children and adolescents three months after initial HRT or ERP treatment. About half of the participants followed a cross-over design where follow-up evaluations were obtained three months after the additional training. The study showed that the acute improvements were maintained. Interpretations were confounded by the cross-over design, and the authors stated that they were unable to draw conclusions about the long term effects of ERP or HRT separately. The intermediate effect of CBIT in children has been evaluated in a randomised, controlled trial [6]. It was shown that 20 out of 32 (62.5%) positive responders continued to show benefit in the follow-up period of 6 months. In a study from 2018, long-term outcome (12 months) was examined in HRT group versus educational group setting [15]. Over the follow-up period, both groups showed a continued improvement in total tics score and especially in motor tic score. In an internet-based study, young people with chronic tic disorder were treated with therapist-guided and parent-guided internet-delivered programmes (BIP TIC HRT or BIP TIC ERP). Patients in both groups maintained their therapeutic gain in the 12 months follow-up period [16].

In adults, Deckersbach et al. [9] examined the intermediate efficacy of HRT in comparison with supportive psychotherapy. The adult patients were re-assessed after 6 months and it was shown that the initial reduction in tic severity was stable 6 months after ended HRT treatment. Comparably, improvements in life-satisfaction and psychosocial functioning remained stable in both the HRT and the supportive group. In another study from 2012 [7], Wilhelm et al. re-assessed adult patients three and 6 months post-treatment. All participants showed a positive response to initial CBIT or supportive treatment. At 6 months, 80% showed a continued positive response suggesting that the improvements were stable over time. Correspondingly, Wilhelm et al. re-assessed adults with chronic tic disorder 10 months after acute treatment. They showed that the acute improvement was maintained with regard to tic severity and impairment [8].

Overall, the studies have looked at the intermediate and long-term treatment effect after individual treatment with HRT and CBIT. Evaluation of long-term outcomes after an initial treatment with ERP is lacking and only one study has looked at the long-term effect after group treatment of children and adolescents with a tic disorder [17].

In an open randomised controlled clinical trial, Nissen et al. [11] examined the effectiveness of a treatment manual combining HRT and ERP in an individual or a group setting. Significant reductions in Total Tic Severity Score (TTS) and in Functional Impairment (FI) as measured by the Yale Global Tic Severity Scale (YGTSS) [18] were shown after eight sessions in both individual and group settings. A total of 66.7% of the participants were considered responders. Internalising symptoms predicted a lesser decrease in FI, whereas the occurrence of obsessive–compulsive symptoms was associated with a larger decrease in TTS [19].

The aims of the present study were to report on the re-assessment 12 months post-treatment of the combined treatment with HRT and ERP to identify possible latent class trajectories of TTS, and to identify possible predictors of the class membership.

## Methods

### Participants and treatment

The randomised clinical trial is described in [11]. In brief, children and adolescents (N = 102, age 9–17 years) were referred to the Department of Children and Adolescent Psychiatry, Aarhus University hospital, Denmark (described in the included flowchart). Children and adolescents were assessed using a modified version of the *Schedule for affective disorders and schizophrenia for school-age children–present and lifetime version (K-SADS-PL)* ([20]) administered to the parents and child/adolescent separately. The K-SADS-PL information was used to confirm a primary diagnosis of chronic tic disorder, to diagnose any comorbidities such as obsessive compulsive disorder, affective disorder, psychosis, anorexia, anxiety disorders, planning difficulties (ICD-10 code: DF83.9), and to ensure that none of the exclusion criteria were met [11]. The inclusion and exclusion criteria were chosen as to ensure that the study would be representative of clinical practice. Exclusion criteria included disorders that required immediate treatment: psychotic disorder, primary severe depression, suicidal ideation or attempts, primary severe anorexia nervosa. Furthermore, children and adolescents were excluded if their IQ was below 70, they had a life-time diagnosis of pervasive developmental disorder, or if they had been treated with HRT or ERP during the last 6 months.

The children and adolescents (N = 59, age 9–17 years) with a primary diagnosis of either Tourette syndrome or chronic motor or vocal tic disorder as described in the WHO ICD-10 diagnostic criteria and the Diagnostic and Statistical Manual of Mental Disorders, Fourth edition, Text Revision, and of moderate or greater severity corresponding to a total score on the Yale Global Tic Severity Scale (YGTSS) [18] higher than 13 (higher than nine if only motor or vocal tics were described) [6], and who did not meet the exclusion criteria were offered manualised treatment constituted by a combination of habit reversal training (HRT) and exposure response prevention (ERP) either as an individualized treatment or in a group setting [11]. The therapeutic treatment was based on the newly developed manual adapted by the individual treatment manuals by Woods et al. [21] and Verdellen et al. [22]. The manual described a nine-session therapy for either individual or group treatment. In both settings, participants trained in HRT for 2 sessions, and ERP for two sessions. In the following sessions, the participants trained in both treatment modalities depending on the presented tic symptom. There were 2 weeks between the first six sessions. Hereafter, there were 3 weeks between the next three sessions, and between session 8 and the delayed booster session 9, there were 8 weeks. The parents participated in the last 15 min of each individual session, or in the end of the second, fourth, eighth and ninth session in the group setting. The total number of the participants who completed all sessions was 54. They were all contacted 6 and 12 months after the 8th session. Completers of follow-up were defined as participants who were assessed at 12 months follow-up.

The study was approved by the National Ethical Committee (1-10-72-216-15) and the Danish Data Protection Agency (1-16-02-490-15), registered 12 October 2015. Oral and written information was given to parents and patients, and written consents from patients over 15 years of age and parents were received.

### Evaluations and assessments

Evaluations were conducted at the 8th session (acute outcome), after the 9th session (8 weeks delayed booster session), and 6 and 12 months after the 8th session. The main outcome measures were TTS (motor score + vocal score) and FI as evaluated by YGTSS, and a positive therapeutic improvement, defined as a more than 25% reduction in severity scores [23]. Evaluations of treatment response were made by an independent evaluator who was not blinded to the treatment allocation, yet not involved in the treatment of the patient, and blinded to any previous evaluations.

The YGTSS is a clinician-administered semi-structured interview including a checklist of all tics present in the past week. It covers five dimensions divided into ten items including the number, frequency, intensity, complexity and interference of motor and vocal tics. Furthermore, it includes a separate evaluation of the functional impairment. The scores are summed to yield separate motor and vocal tic scores (0–25) and a combined total tic score (0–50). The functional impairment scale (range 0–50) rates the tic-related disability over the past week [18, 23].

The parents and the children/adolescents were furthermore asked to complete Screen for Child Anxiety Related Emotional Disorders (SCARED) [24], the Mood and Feelings Questionnaire (MFQ) [25], the Premonitory Urge Scale (PUTS) [26] and Beliefs About Tics Scale (BATS) [27]. Furthermore the parents completed SCARED, the MFQ, Child Behaviour Checklist (CBCL) [28], and Sensory Profile [29].

SCARED [24] includes separate versions for parents and the child/adolescent. Using 41 items rated on a three-point scale, the questionnaire assesses the occurrence of anxiety symptoms based on DSM-IV. Scores range from zero to 82.

MFQ assesses the occurrence of depressive symptoms, using 13 items rated on a three-point scale [25]. Scores range between 0 and 26, where high scores indicate a severe functional impairment.

PUTS is a short self-reporting scale with nine items [26]. It measures the tic-related premonitory urge. The scale was developed by D. Woods and colleagues, US. For this project, the scale was translated into Danish by the principal investigator (J. Nissen). After a re-translation into English, the scale was approved by D. Woods, US.

BATS is a self-reporting scale with 20 items developed to assess the different beliefs children and adolescent experience in relation to tic symptoms and to suppressing their tic symptoms [27]. The scale was developed by T. Steinberg, A. Apter and colleagues, Israel. For the present study, the scale was translated into Danish by the principal investigator (J. Nissen). After a re-translation to English, the scale was approved by Dr. Steinberg, Schneider Children`s Medical Center, Israel.

CBCL is a parent questionnaire evaluating behavioral and emotional problems in children and adolescents [28]. The questionnaire is used in the age range 6–18 years. CBCL has 113 items rated on a three-point scale. The results are depicted both in a total problem scale and several subscales.

The Dunn Sensory Profile 2 was used to access any sensory challenges. It is a collection of questionnaires for different age groups [29]. The aim of the questionnaires is to assess children's responses to commonly occurring sensory events and to evaluate the ability to process the sensorimotor impressions. The questions are grouped into three main areas: sensory processing, sensory modulation and behavior, and emotional response.

### Statistics

Two-sample t-tests were used to compare outcome scores at baseline and after 8 weeks of treatment of completers and non-completers of follow-up, between individual and group setting at 6 and 12 months, and between therapeutic improvers and non-therapeutic improvers at 12 months. A positive therapeutic improvement was defined as a more than 25% reduction in severity scores [24]. The association between group setting and TTS and FI over time was assessed using mixed linear regressions models with a random intercept to account for the repeated measurements within subject. The association between TTS and BATS, PUTS, SCARED and MFQ over time were assessed using the latter approach.

Response trajectories based on TTS score at all time points were estimated using a semi-parametric group-based trajectory model (GBTM) for repeated measurements [30, 31]. The GBTM was fitted using the Stata Plugin TRAJ for estimating group-based trajectory models. TTS score were modelled using the censored normal distribution [31, 32].

The models were tested with one to four trajectory groups to identify the optimal number of groups to fit the data. In the initial model, trajectory group orders were examined as quadratic models. Each participant was assigned exclusively to the trajectory group for which s/he had the highest posterior probability of group membership. The best fitting model was selected on the basis of the following criteria: (1) a group size of at least 5% of the analysed population, (2) the highest possible Bayesian Information Criterion (BIC) and Akaike Information Criterion (AIC), (3) a minimum average posterior probability (AvePP) of 0.7 for group membership, (4) an odds of correct classification (OCC) above 5, and (5) agreement between the estimated probability of group membership and the proportion assigned to each group according to the maximum posterior probability assignment rule.

When criterion 1 (group size) was fulfilled, the best fitting model was identified on the basis of a simultaneous evaluation of the remaining criteria. Once the best fitting model was chosen, the shape of the trajectory of each group was investigated and it was found that a cubic shape resulted in the best fitting model. GBTM uses the maximum likelihood method to estimate model parameters. This will generate asymptotically unbiased parameter estimates assuming the data are missing at random (MAR) [32].

Odds ratios (ORs) and 95% CIs were determined for the association between age, gender, group setting, ADHD, planning difficulties, hypersensitivity, having OCD, Anxiety, PUTS, BATS and CBCL and response trajectory group using logistic regression models. The lesser responder trajectory group was used as reference in the analyses.

The association between BATS and PUTS and trajectory group over time was assessed using mixed linear regressions models with a random intercept to account for the repeated measurements within subject. The between trajectory group differences at all time points were assessed using chi square statistics. All analyses were undertaken using Stata 16.1 (StataCorp, Texas, USA).

**Figure:**
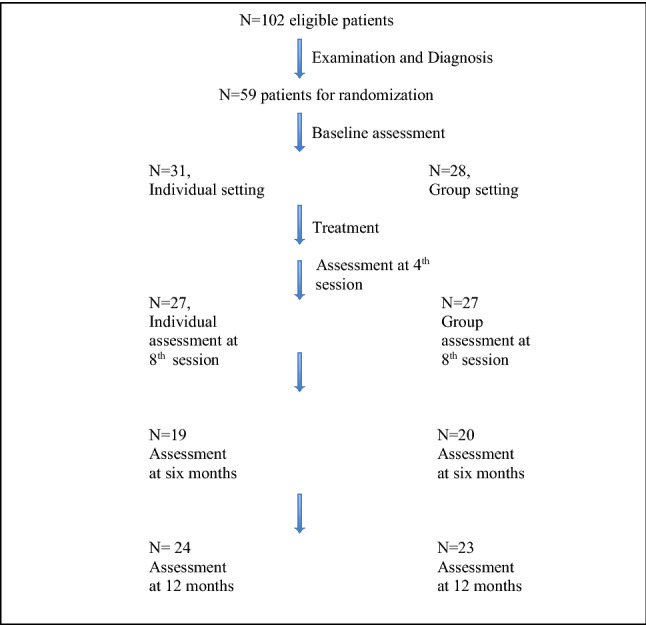
Flowchart of the combined treatment and follow-up of children and adolescents with CTD

## Results

### Description of the completers at 12 months

A total of N = 39 participants (72% of those evaluated at session eight; male N = 25, 53.2%) and N = 47 participants (87% of those evaluated at session eight; male N = 29, 61.7%) completed evaluations at 6 and 12 months, respectively. There was no significant difference in tic severity or age at baseline in those completing the evaluation compared to those who dropped out of the study (Table 1).

**Table 1:** Comparison of completers and non-completers at 12 months

	Completer of follow-up N = 47, mean (SD)	Non-completer of follow-up N = 7, mean (SD)	p-value
Baseline TTS	23.52 (6.56)	24.12 (7.24)	0.82
8th session TTS	14.60 (5.91)	18.57 (0.11)	0.11
Baseline FI	25.49 (8.04)	27.86 (8.05)	0.47
8^th^ session FI	11.91 (6.88)	15.71 (8.38)	0.19
Age	12.09 (2.21)	12.43 (2.51)	0.71

Mean (SD),* p < 0.05

### Outcome at 6 and 12 months

Of those who completed follow-up at six (N = 39) and 12 months (N = 47), TTS were 14.03 ± 8.27 and 12.72 ± 5.94 and FI scores were 12.18 ± 9.23 and 10.13 ± 7.42, respectively. There were no significant difference between individual and group setting (Table 2).

**Table 2:** TTS and FI at 6 and 12 months evaluated after individual or group therapy

	Individual mean (SD)	Group mean (SD)	p-value
TTS 6 months	12.63 (7.73)	15.35 (8.73)	p = 0.31
TTS 12 months	12.67 (6.19)	12.78 (5.81)	p = 0.95
FI 6 months	10.56 (8.73)	13.57 (9.64)	p = 0.32
FI 12 months	8.75 (7.11)	11.57 (7.63)	p = 0.20

Mean (SD), *p < 0.05

**Fig. 1:**
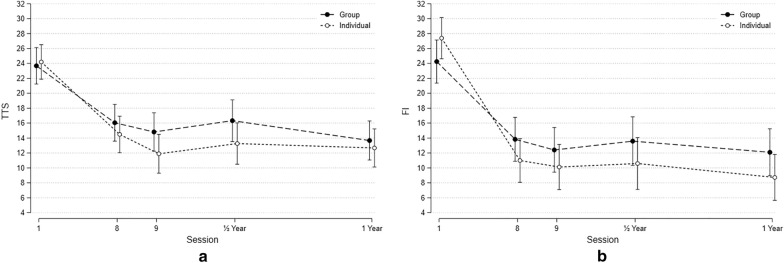
**a** The figure shows the course of TTS score (Y-axis) from baseline to 12 months follow-up (Mean and SD. **b** The figure shows the course of FI score (Y-axis) from baseline to 12 months follow-up (Mean and SD)

In a mixed model, it was shown that the initial improvement in both individual and group setting was maintained throughout the follow-up period (Fig. 1). There were no significant differences between individualised and group treatments (between group differences: TTS: 0.09, p = 0.77; FI: 0.04, p = 0.84).

Since there were no significant differences between the two methods of delivery the following paragraphs describe the merged results from the groups.

### Therapeutic improvers versus non-therapeutic improvers at 12 months

Of all participants completing the 12 months evaluation, 74.4% (N = 35) were considered therapeutic improvers (TTS) defined as a more than 25% reduction in severity scores (TTS). Of all participants who showed a positive response in TTS after acute treatment, 61.1% (N = 33) were considered therapeutic improvers at 12 months. Of the participants who were non–therapeutic improvers after acute treatment, 50% (N = 7) showed a positive response after 1 year, whereas N = 5 participants did not reach responder status again at 12 months.

Comparing the therapeutic improvers and non-therapeutic improvers at 12 months, neither PUTS score, BATS score, CBCL, SCARED score or MFQ score differed. However, the therapeutic improvers group showed a higher TTS score at baseline compared to the non- therapeutic improvers (Table 3, Additional file 1: Table S1).

**Table 3:** Multivariate analyses of baseline characteristics

	OR (CI), p
TTS at baseline	1.19 (1.06–1.33), p 0.003*
Gender (male vs female)	0.73 (0.25–2.13), p 0.57
Age	0.84 (0.66–1.07), p 0.16
Individual versus group	0.63 (0.22–1.81), p 0.39
ADHD	1.31 (0–44–3.90), p 0.63
Planning difficulties	2.13 (0.43–10.54), p 0.35
Hypersensitivity	1.75 (0.40–7.58), p 0.45
OCD	0.70 (0.12–4.19), p 0.70
Anxiety	0.97 (0.24–3.87), p 0.96
PUTS	0.98 (0.89–1.09), p 0.66
BATS	1.01 (0.96–1.08), p 0.62
CBCL	1.02, (1.00–1.04), p 0.08

OR (CI), *p < 0.05

### Associations between TTS and BATS, PUTS, SCARED and MFQ at all time-points

**Fig. 2:**
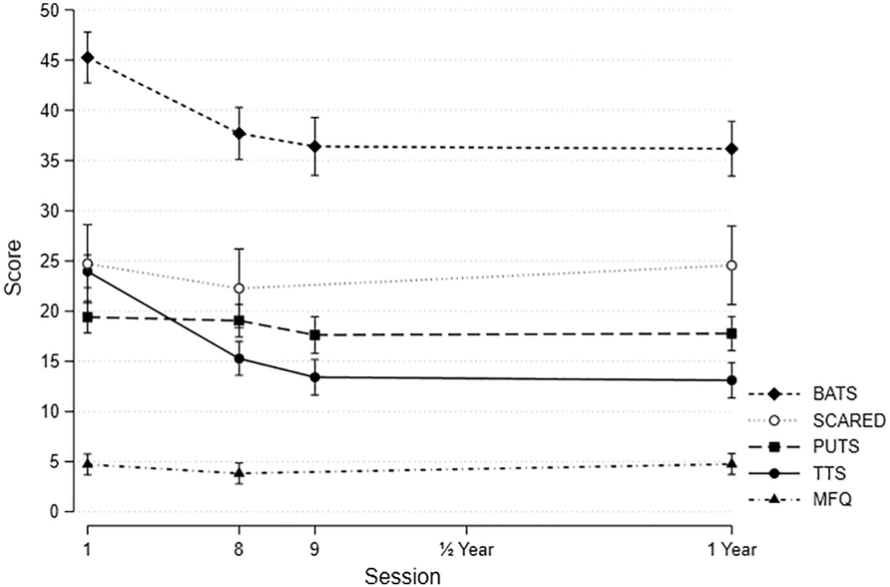
The figure shows the course of BATS, SCARED, PUTS, MFQ and TTS (Y-axis) from baseline to 12 months follow-up (Mean and SD)

There was a significant positive association between the reduction of TTS and the reduction in the BATS score from baseline to follow-up (12 months) (coef. 0.21 (0.11–0.30), p = 0.0001). A similar, but weaker positive association was seen for MFQ (coef 0.34 (0.04–0.63), p = 0.02). (Fig. 2). There were no significant associations between TTS scores and PUTS or SCARED scores.

### Symptom severity (TTS) trajectory classes

**Fig. 3:**
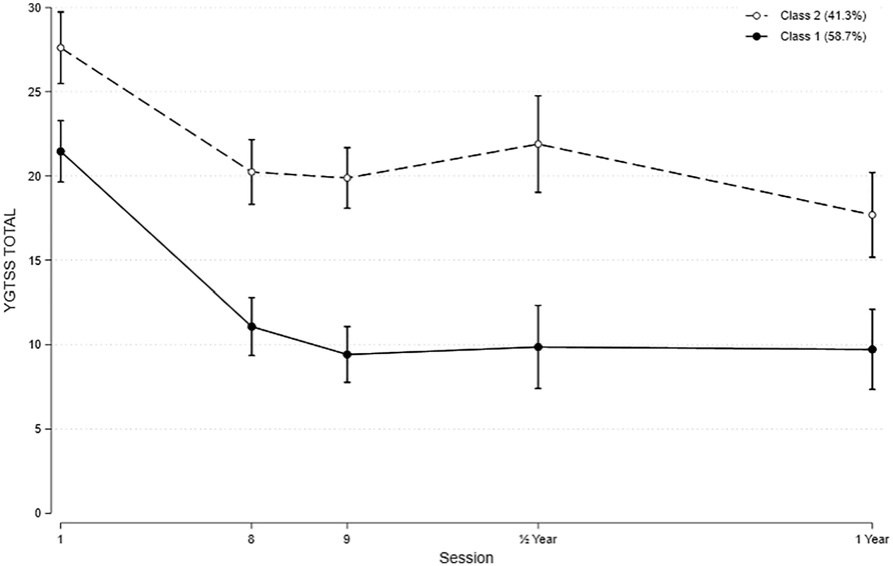
The best fit for symptom severity (TTS) trajectory classes

The best fit was a model with two classes and a cubic curve (Fig. 3). (BIC − 795.44, AIC − 777.94, AvePP 0.95, OCC 13.10, est_p 0.59 (class 1), AvePP 0.94, OCC 23.6, est_p 0.41 (class 2)).

Class 1 (Major responder) comprised 58.7% of the participants and showed a significant initial effect (p = 0.001), which was stable across the follow-up period.

Class 2 (Lesser responder) included 41.3% of the participants and was characterised by a significant lesser reduction during acute treatment. The reduction in TTS compared to baseline was still significant (p = 0.001). The initial acute outcome evaluated at the 8th session also stabilised in this class.

### Characteristics of symptom severity trajectory classes

Additional file 2: Table S2, which presents the demographic baseline characteristics in the two classes.

Multivariate analyses showed that a high baseline TTS significantly increased the likelihood of being in class 2. Furthermore, there was a tendency that having ADHD, planning difficulties, or hypersensitivity also increased the likelihood of being in class 2. Other factors did not show any notable association (Table 3).

**Fig. 4:**
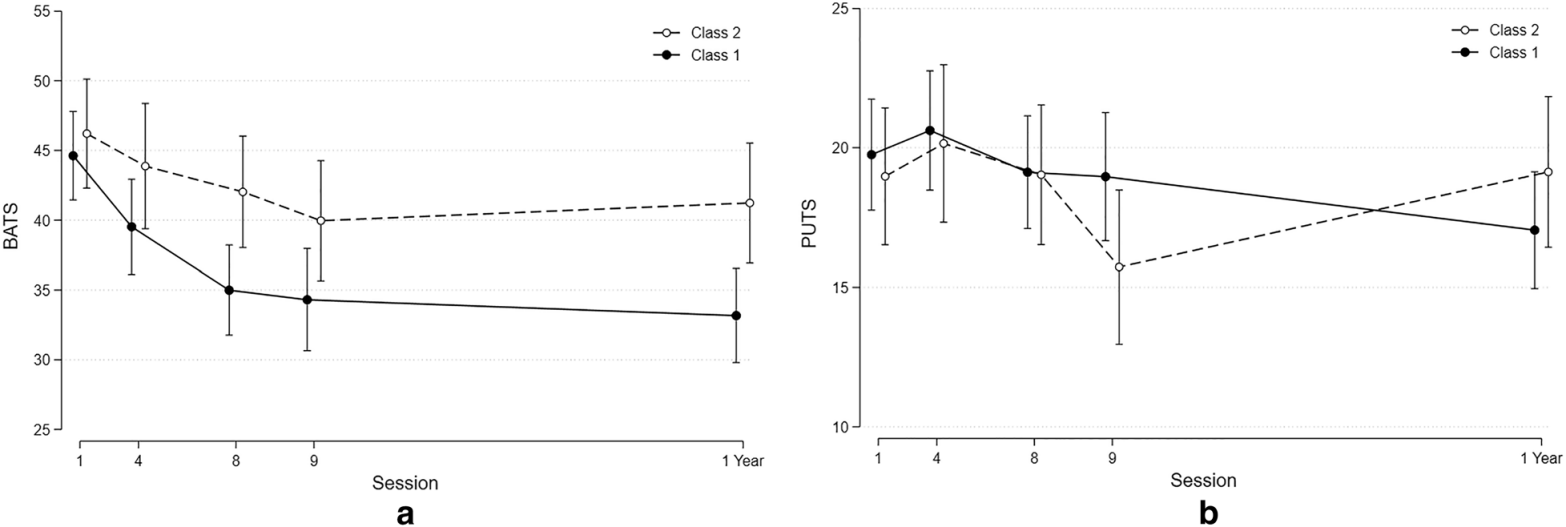
**a** BATS scores distributed in accordance to the symptom severity (TTS) trajectory classes. **b** PUTS scores distributed in accordance to the symptom severity (TTS) trajectory classes

The two classes did not differ in the BATS score at baseline. However, class 1 participants showed a significant greater reduction in BATS score which was sustained throughout the follow-up period (Table 4, Fig. 4a, b). No difference was seen for PUTS scores.

**Table 4:** Between group differences in BATS and PUTS score at all time-points

	Between group difference mean (SD)	Chi2 (1) (p)
BATS scores		
0–6 weeks (4th session)	2.77 (− 2.84–8.38)	0.94 (p = 0.33)
0–16 weeks (8th session)	5.45 (0.32–10.58)	4.33 (p = 0.04)*
0–24 weeks (9th session)	4.07 (− 1.52–9.65)	2.03 (p = 0.15)
0–68 weeks (1 year)	6.47 (1.04–11.91)	5.45 (p = 0.02)*
PUTS scores		
0–6 weeks (4th session)	0.32 (− 3.23–3.86)	0.03 (p = 0.86)
0–16 weeks (8th session)	0.69 (− 2.57–3.95)	0.17 (p = 0.68)
0–24 weeks (9th session)	− 2.46 (− 6.06 to − 1.14)	1.80 (p = 0.18)
0–68 weeks (1 year)	2.87 (− 0.57 to − 6.31)	2.67 (p = 0.10)

Mean, SD, Chi2 (1), *p < 0.05

## Discussion

Both separately and in combination, HRT and ERP are known to be effective therapeutic interventions towards chronic tics disorders. This has been shown as immediate outcome [6–11], and in intermediate and long-term follow-up studies [7, 9, 10, 15–17]. Thus, for both adolescents and adults more than 80% of available responders maintained a positive response at intermediate and long-term follow-up. In the present study, 87% of the participants evaluated at the 8th session were accessible for re-evaluation at 12 months after combined treatment with HRT and ERP. For these adolescents, it could be shown that the overall YGTSS TTS and FI scores were maintained at a low level, and 74.4% were considered responders (TTS). These results were comparable for the individual and group setting. The result suggests a continuous positive response of a combined training in both an individual and group setting, that is comparable to the results shown in other studies [10, 15–17]. The previously presented studies have looked at the intermediate and long-term treatment effect after individual treatment with HRT and CBIT. Evaluation of long-term outcomes after an initial treatment with ERP is lacking, and only one study has looked at the long-term effect after group treatment of children and adolescents with a tic disorder. The present study presents the long-term effect of combined HRT and ERP treatment thereby adding to the existing literature on long-term outcome of therapeutic treatments of chronic tic disorders. Together with the other studies on behaviour treatment of chronic tic disorder, the importance of having access to therapeutic interventions is emphasized, which may be HRT and ERP separately or in combination, and offered as an individual or as a group intervention.

Nissen et al. [19] has previously shown that Beliefs About Tics Scale (BATS) [21] scores at the 8th session moderated the perceived FI [19]. In the present study, there was a significant positive association between the BATS scores and TTS over the follow-up period also pointing towards an importance of persistent thoughts. Distress, negative thought, sadness, and feeling stressed are often described to aggravate tic severity. This may also include the child`s interpretation and thoughts of their tics. Thus, as previously suggested [11], it could be important to include cognitive elements in the treatment procedures. Of interest, a weaker, but positive association was also seen for mood and feelings questionnaire (MFQ) supporting the importance of a child`s mood. When evaluating immediate outcome, we have previously shown that a high scores on the MFQ favoured individual treatment [19]. The present study suggests, that mood also may influence long-term outcome independently of the setting.

To our knowledge, the latent class post-treatment trajectory analyses have not previously been performed on chronic tic data. The analysis is a supplement to the reporting of long-term tic severity and helps to identify groups of patients based on similarities in tic severity. Based on the entire follow-up period, we identified two classes where high baseline severity increased the likelihood of being in the less responder class. Similar, but only as a trend, having ADHD, planning difficulties, or hypersensitivity increased the risk of a lesser response both as an acute outcome and over a 12 months follow-up period. Thus, children and adolescents evaluated with a high TTS score, or diagnosed with ADHD, planning difficulties, or hypersensitivity may need a more intensive tic treatment as to achieve as significant long-term treatment outcome. The lesser treatment effect can be seen early in the treatment suggesting a benefit of regular evaluations during treatment. Based on the two classes, it was furthermore shown that BATS scores were reduced significantly more in class 1 compared to class 2 independently of baseline BATS score. This finding supports that there is a connection between the treatment outcome and the change in the participants thoughts about their tics. Only few other studies have looked at predictors of follow-up outcomes. Lowe et al. [33] showed an improvement for the majority of participants, but they did not find any robust predictors of follow-up outcomes. On the contrary, Groth [34] showed that childhood tics, OCD, and ADHD severity were predictors for long-term symptoms of the respective diagnoses. The participants had received different treatments both including medication and therapy. The study showed that tics tend to fade in severity although comorbid conditions like OCD and ADHD may affect the course, not least the continued co-occurrence of obsessions/compulsions and ADHD related symptoms. To out knowlegde, no previous study has looked at predictors for long-term therapeutic treatment outcome in adolescent chronic tic disorders.

## Conclusions

The present study adds to the existing evidence that HRT and ERP are effective long-term treatment modalities towards chronic tics disorders in adolescents both as an individual treatment, and treatment in groups. The importance of being attentive to the child`s thoughts about their tics is stressed. Factors like baseline tics severity, and the occurrence of ADHD, planning difficulties and hypersensitivity may affect the long-term outcome increasing the risk of belonging to the group with the poorest outcome. This effect will show early in the course emphasising a thorough baseline examination and a close follow-up of treatment effect.

## Limitations

The results of the study should be seen in light of the limited number of participants. The trajectory analysis was therefore conducted merging the individual and group setting.

## Supplementary Information

**Additional file 1: Table S1.** Comparing therapeutic improvers and non-therapeutic improvers at 12 months, mean (sd), p (*p < 0.05).

**Additional file 2: Table S2.** Baseline characteristics of the two classes, p (*p<0.05).

## Data Availability

The datasets generated and/or analysed during the current study are not publicly available due to Danish law. JBN and AHC confirm to have full access to all the data in the study, and to take responsibility for the integrity of the data and the accuracy of the data analysis.

## Abbreviations

HRT: Habit reversal training
ERP: Exposure response prevention
TTS: Total Tic Severity score
YGTSS: Yale global tic severity scale
BATS: Beliefs about tics scale
BIP TIC HRT or BIP TIC ERP: Therapist-guided and parent-guided internet-delivered programmes
K-SADS: Schedule for affective disorders and schizophrenia for school-age children–present and lifetime version
SCARED: Screen for child anxiety related emotional disorders
MFQ: The mood and feelings questionnaire
PUTS: The premonitory urge scale
CBCL: Child behaviour checklist
