# Supplementary material for: One-year outcome of manualised behavior therapy of chronic tic disorders in children and adolescents
Source: Child Adolesc Psychiatry Ment Health. 2021 Feb 20;15:9. doi: 10.1186/s13034-021-00362-w (PMC7897385; doi:10.1186/s13034-021-00362-w)
Supplement: Supplementary file 1 — Additional file 1: Table S1. Comparing therapeutic improvers and non-therapeutic improvers at 12 months, mean (sd), p (*p < 0.05). [file 13034_2021_362_MOESM1_ESM.docx]

|  | Responder at twelve months (N=35) | Non-responder at twelve months (N=12) | p-value |
| --- | --- | --- | --- |
| TTS baseline | 24.63 (6.44) | 20.25 (6.00) | 0.04* |
| TTS 8^th^ session | 14.26 (5.98) | 15.58 (5.85) | 0.51 |
| FI baseline | 24.97 (7.69) | 27 (9.18) | 0.46 |
| FI 8^th^ session | 11.71 (7.37) | 12.5 (5.43) | 0.74 |
| PUTS baseline | 19.39 (5.76) | 19.82 (6.64) | 0.84 |
| BATS baseline | 44.21 (9.56) | 49.36 (9.28) | 0.13 |
| Scared patient baseline | 21.72 (14.69) | 22.2 (10.84) | 0.93 |
| Scared parent baseline | 22.18 (15.62) | 12.58 (7.67) | 0.05 |
| MFQ patient baseline | 4.36 (3.66) | 4.27 (3.66) | 0.94 |
| MFQ parent baseline | 5.67 (5.26) | 3 (2.09) | 0.10 |
| CBCL baseline | 8.79 (8.44) | 5.17 (5.52) | 0.18 |
| Sensitivity baseline | 0.8 +/- 0.41 | 0.75 +/- 0.45 | 0.72 |

Table S1 Comparing non-responders and responders at twelve months, mean (sd), p (*p<0.05) Suppl.mat.
