# Supplementary material for: One-year outcome of manualised behavior therapy of chronic tic disorders in children and adolescents
Source: Child Adolesc Psychiatry Ment Health. 2021 Feb 20;15:9. doi: 10.1186/s13034-021-00362-w (PMC7897385; doi:10.1186/s13034-021-00362-w)
Supplement: Supplementary file 2 — Additional file 2: Table S2. Baseline characteristics of the two classes, p (*p<0.05). [file 13034_2021_362_MOESM2_ESM.docx]

|  | Class 1 | Class 2 | p-value |
| --- | --- | --- | --- |
| Baseline TTS | 21.46+/-1.19 | 27.58+/-4.55 | p=0.0004 |
| Baseline FI | 25.4+/-8.71 | 26.63+/-6.88 | p=0.57 |
| PUTS baseline | 19.97+/-5.77 | 19.29+/-5.45 | p=0.67 |
| BATS baseline | 44.59+/-9.34 | 45.90+/-10.05 | p=0.63 |
| Scared patient baseline | 21.94+/-13.05 | 27.85+/-16.19 | p=0.15 |
| MFQ patient baseline | 4.25+/-3.46 | 5.43+/-4.36 | p=0.28 |
| Sensitivity | 0.80+/-0.41 | 0.88+/-0.34 | p=0.46 |

Table S2 baseline characteristics of the two classes, p (*p<0.05) Suppl. Mat.
